# Professores que Foram Exemplos Acadêmicos

**DOI:** 10.36660/abc.20201161

**Published:** 2021-06-08

**Authors:** Charles Mady

**Affiliations:** 1 Universidade de São Paulo Faculdade de Medicina Unidade Clínica de Miocardiopatias e Doenças da Aorta São PauloSP Brasil Universidade de São Paulo Faculdade de Medicina - Unidade Clínica de Miocardiopatias e Doenças da Aorta , São Paulo , SP – Brasil

**Keywords:** Educação Médica, Docentes, Sociedades Médicas, Hospitais Públicos, Faculdades de Medicina

Vivemos, ultimamente, em uma sociedade extremamente competitiva e individualista – características que tornam erros e defeitos mais aparentes. A pandemia na saúde, além das endemias de corrupção na política e no tecido social, levou-nos a uma tempestade perfeita. Seus estragos são de difícil solução, pois vários fatores são crônicos, enraizados, quase atávicos, presentes em todos os segmentos da sociedade. A solução chama-se educação. Como diz Paulo Freire, educado e respeitado, mas criticado por deseducados, a educação modifica os indivíduos, fazendo-os evoluir. Estes modificam a sociedade. É, portanto, projeto a longo prazo. ^1 - 3^

Como já comentei em vários artigos na imprensa, temos recursos humanos de elevado nível moral e intelectual. Mas, com o atual sistema político, maligno, constituído por corporativismos e fisiologismos sólidos, que mandam, comandam e desmandam, bloqueia-se a evolução. Esse sistema criminoso retira de cena boa parte dos bem-intencionados. Alguns conseguem vencer, superando os obstáculos, e fazem história, e deixam marcas positivas, tornando-nos mais educados e preparados para levar adiante os seus ensinamentos. Reconhece-se a qualidade pelos herdeiros, levando adiante a boa escola. O Instituto do Coração (InCor) é exemplo a ser seguido, tendo sido recentemente qualificado como um dos melhores hospitais públicos do mundo. É orgulho para nosso país.

Na Medicina, que é a minha seara, encontrei o que há de melhor e de pior referente ao ser humano. Quando a busca desenfreada por prestígio e dinheiro ocupa a mente de pessoas moralmente fracas, os maus exemplos se apresentam. Essa doença está aumentando em minha profissão, seguindo os passos de outros setores da sociedade. A palavra *caráter* está aos poucos sendo erodida. No entanto, com otimismo, vamos lembrar aqueles que seguiram a cartilha de Paulo Freire, formando e educando no bom caminho, levando nossa Medicina ao reconhecimento internacional. Tive exemplos memoráveis que marcaram minha formação, dando lições de humildade e de sabedoria. Não perseguiam holofotes, pois a profissão era um fim, e não um meio. Tomo a liberdade de citar alguns, com os quais tive relação direta.

No início de meu curso, em 1965, na Faculdade de Medicina da Universidade de São Paulo (USP), fui aluno de Odorico Machado de Sousa, sempre presente, que conseguia transformar o difícil curso de anatomia em algo prazeroso. Permanecia – ele e sua equipe – diariamente conosco, pronto a responder a qualquer dúvida. Era exigente, e nos ensinou a enxergar sua matéria de forma construtiva, valorizando-a e relacionando-a às outras disciplinas. Transformou a rejeição em aprendizado. Portanto, nos educou. Em meu quarto ano, conheci Luiz Décourt e Euryclides de Jesus Zerbini, acadêmicos na definição exata da palavra. Fizeram escola internacional, com incontáveis discípulos, que se tornaram líderes em seus locais de origem, e em nossa universidade. Faziam questão de, pessoalmente, ensinar, mantendo socraticamente proximidade com os alunos. A finalidade primordial era a faculdade. Construíram, com suas equipes por eles educadas, o InCor, elevando ainda mais o conceito da USP. Deixaram uma herança reverenciada até hoje. Foram reais Professores, com P maiúsculo. Seus alunos têm orgulho de terem sido seus discípulos.

Décourt foi sucedido por Fulvio Pileggi, que dedicou todo o seu prestígio e força política para engrandecer ainda mais o InCor. Portador de um caráter exigente, solicitava pessoalmente doações à sociedade, pois a verba pública era insuficiente para realizar seu projeto. Estimulava a assistência, o ensino e a pesquisa, sabendo que esses eram os determinantes de qualidade acadêmica. Cobrava pessoalmente resultados, com presença constante no InCor. Deixava a porta sempre aberta. Sua rigidez escondia um coração incapaz de guardar mágoas. Macruz e Tranchesi, também discípulos de Décourt, foram seus colaboradores, acadêmicos do mais elevado nível, e que também muito nos ensinaram. Hoje, seus herdeiros estão à altura dos mestres. ^4^

Em viagens por este país, tive a oportunidade de conhecer centros universitários que me impressionaram pelo enorme potencial cultural. O falecido Prof. Pareto, da Universidade Federal Fluminense (UFF), foi exemplo de humildade e sabedoria, tendo muito me marcado. Fez escola, ensinando a ver, ouvir e entender humanamente o paciente, antes de exames e telas. Formou herdeiros brilhantes, que o sucederam. Reconhece-se o mestre por seus produtos.

Quantos há mais, pouco conhecidos, e muitas vezes pouco valorizados, espalhados por aí? Temos que aprender a reconhecer valores e méritos. Esses poucos exemplos servem para mostrar que podemos ter um futuro melhor com vontade política, partindo de dirigentes educados e determinados. Os mestres em todas as matérias estão aí, na difícil tarefa de educar. Aliviem os obstáculos. Deixe-os mostrar seus valores. Temos que aprender a reconhecê-los e cultuá-los. Como costumo dizer, quem não cultua os Mestres jamais será um Mestre.

## References

[1] 1. Mady C. Medical education in Brazil. Arq Bras Cardiol.2009;93(4):e70-1, e58-9. 10.1590/s0066-782x2009001000026 19936447

[2] 2. Mady C. A saúde de nossas universidades.(estadão.com.br) (internet) [Citado em 2021 apr 10] Disponível em :https:/opniao.estadao.com.br/noticias/espaco-aberto a –saude-de-nossas-universidades,70003109850

[3] 3. Mady C. As sociedades médicas e as universidades publicas. Arq Bras Cardiol.2019;112(3):317-8. 10.5935/abc.20180241 PMC6424047 30916195

[4] 4. Mady C. Luiz V Décourt, ícone do humanismo científico. Clinics. 2007;62(4):375-6. 10.1590/s1807-59322007000400001 17823697

