# Supplementary material for: Professores que Foram Exemplos Acadêmicos
Source: Arq Bras Cardiol. 2021 Jun 8;116(6):1172–3. [Article in Portuguese] doi: 10.36660/abc.20201161 (PMC8288535; doi:10.36660/abc.20201161)
Supplement: Translation

# Translation: Teachers who were Academic Examples

**Authors:** Charles Mady

**Affiliations:** 1 Brasil Universidade de São Paulo Faculdade de Medicina - Unidade Clínica de Miocardiopatias e Doenças da Aorta, São Paulo, SP – Brasil

**Keywords:** Education,Medical, Faculty, Societies,Medical, Hospitals, Public, Schools, Medical

We currently live in an extremely competitive and individualistic society, which makes mistakes and defects more apparent. The health pandemic, in addition to the endemics of corruption in politics and the social fabric, has led us to a perfect storm. Its damage is difficult to solve, as several factors are chronic, ingrained, almost atavistic, and are present in all segments of society. The solution is called education. As Paulo Freire says, educated and respected, but criticized by the uneducated, education changes individuals, making them evolve. These change society. It is, therefore, a long-term project.^1 - 3^

As I have commented in several articles in the press, we have human resources of high moral and intellectual level. However, with the current political system, malignant, consisting of corporatism and solid physiologisms, which dispute, command, and disengage, evolution is blocked. This criminal system removes most of the well-meaning people from the scene. Some manage to win by overcoming obstacles. Moreover, they make history. In addition, they leave positive marks, making us more educated and prepared to carry out their teachings. Quality is recognized by the heirs, carrying out the good school. InCor is an example to be followed; it has been recently qualified as one of the best public hospitals in the world. It is a source of pride for our country.

In Medicine, which is my field, I found the best and the worst, referring to the human being. When the unrestrained search for prestige and money occupies the minds of people who are morally weak, bad examples emerge. This disease is increasing in my profession, following in the footsteps of other sectors of society. The word character is gradually being eroded. However, with optimism, let us remember those who followed Paulo Freire’s booklet, training and educating in the right way, leading our Medicine to international recognition. I had memorable examples, which marked my education, giving lessons of humility and wisdom. They did not chase spotlights, as the profession was an end, not a means. I take the liberty of naming some, with whom I had a direct relationship.

At the beginning of my course, in 1965, at the School of Medicine of USP, I was a student of Odorico Machado de Sousa, always present, who could transform the difficult anatomy course into something pleasurable. He and his team remained with us daily, ready to answer any questions. He was demanding, and taught us to see the subject constructively, valuing it, and relating it to other subjects. He turned rejection into learning. So, he raised us. In my fourth year, I met Luiz Décourt and Euryclides de Jesus Zerbini, academics in the exact definition of the word. They went to international school, had had countless disciples, who became leaders in their places of origin, and in our university. They made a point of personally teaching, remaining Socratically close to the students. The primary purpose was college. They built InCor with their teams, educated by them, and further raised the concept of USP. They left a revered heritage to this day. They were real professors, with a capital P. Their students are proud to have been their disciples.

Décourt was succeeded by Fulvio Pileggi, who dedicated all his prestige and political strength to further aggrandize InCor. With a demanding character, he personally requested donations from society, because public funds were insufficient to carry out his project. He stimulated care, teaching and research, knowing that these were the determinants of academic quality. He personally pursued results, with constant presence in InCor. He left the door always open. His stiffness hid a heart unable to hold sorrows. Macruz and Tranchesi, also disciples of Décourt, were his collaborators, academics of the highest level, who also taught us a lot. Today, his heirs are up to the masters.^4^

On trips around this country, I had the opportunity to meet university centers that impressed me by the enormous cultural potential. The late Prof. Pareto, from the Fluminense Federal University, was an example of humility and wisdom, and marked me a lot. He went to school, teaching to see, listen, and humanely understand the patient, with tests and screens. He educated brilliant heirs who succeeded him. The master is recognized for his products.

How many more, little known, and often undervalued, scattered around? We have to learn to recognize values and merits. These few examples serve to show that we can have a better future with political will, starting from educated and determined leaders. The masters in all subjects are there, in the difficult task of educating. Relieving obstacles. Let them show their values. We have to learn to recognize them and cultivate them. As I say, anyone who does not worship the Masters will never be a Master.
